# Novel Parvoviruses from Wild and Domestic Animals in Brazil Provide New Insights into Parvovirus Distribution and Diversity

**DOI:** 10.3390/v10040143

**Published:** 2018-03-22

**Authors:** William Marciel de Souza, Tristan Dennis, Marcílio Jorge Fumagalli, Jansen Araujo, Gilberto Sabino-Santos, Felipe Gonçalves Motta Maia, Gustavo Olszanski Acrani, Adriano de Oliveira Torres Carrasco, Marilia Farignoli Romeiro, Sejal Modha, Luiz Carlos Vieira, Tatiana Ometto, Luzia Helena Queiroz, Edison Luiz Durigon, Márcio Roberto Teixeira Nunes, Luiz Tadeu Moraes Figueiredo, Robert James Gifford

**Affiliations:** 1 Virology Research Center, Ribeirão Preto Medical School, University of São Paulo,14049-900 Ribeirão Preto, SP, Brazil; marcilio_jorge@hotmail.com (M.J.F.); sabinogsj@usp.br (G.S.-S.J.); felipegmaia@gmail.com (F.G.M.M.); mafarignoli@hotmail.com (M.F.R.); luiz_carlos_vieira@hotmail.com (L.C.V.); ltmfigue@fmrp.usp.br (L.T.M.F.); 2 MRC-University of Glasgow Centre for Virus Research, Glasgow G61 1QH, UK; t.dennis.1@research.gla.ac.uk (T.D.); sejal.modha@glasgow.ac.uk (S.M.); 3 Institute of Biomedical Sciences, University of São Paulo, 05508-900 São Paulo, SP, Brazil; jansentequila@usp.br (J.A.); tatiometto@usp.br (T.O.); eldurigo@usp.br (E.L.D.); 4 Universidade Federal da Fronteira Sul, Passo Fundo, RS 99010-200, Brazil; gacrani@gmail.com; 5 Universidade Estadual do Centro-Oeste, Guarapuava 85015-430, Paraná, Brazil; adriano.carrasco@gmail.com; 6 Faculty of Veterinary Medicine, São Paulo State University, Araçatuba, SP 16050-680, Brazil; lhqueiroz@fmva.unesp.br; 7 Center for Technological Innovations, Evandro Chagas Institute, Ministry of Health, Ananindeua, Pará 67030-000, Pará, Brazil; marcionunesbrasil@yahoo.com.br

**Keywords:** parvovirus, *Parvoviridae*, ssDNA viruses, zoonotic viruses

## Abstract

Parvoviruses (family *Parvoviridae*) are small, single-stranded DNA viruses. Many parvoviral pathogens of medical, veterinary and ecological importance have been identified. In this study, we used high-throughput sequencing (HTS) to investigate the diversity of parvoviruses infecting wild and domestic animals in Brazil. We identified 21 parvovirus sequences (including twelve nearly complete genomes and nine partial genomes) in samples derived from rodents, bats, opossums, birds and cattle in Pernambuco, São Paulo, Paraná and Rio Grande do Sul states. These sequences were investigated using phylogenetic and distance-based approaches and were thereby classified into eight parvovirus species (six of which have not been described previously), representing six distinct genera in the subfamily *Parvovirinae*. Our findings extend the known biogeographic range of previously characterized parvovirus species and the known host range of three parvovirus genera (*Dependovirus*, *Aveparvovirus and Tetraparvovirus*). Moreover, our investigation provides a window into the ecological dynamics of parvovirus infections in vertebrates, revealing that many parvovirus genera contain well-defined sub-lineages that circulate widely throughout the world within particular taxonomic groups of hosts.

## 1. Introduction

Parvoviruses are small, linear and non-enveloped viruses with single-stranded DNA (ssDNA) genomes ~5–6 kilobases (kb) in length [1]. All parvoviruses possess at least two major genes, a non-structural (NS) gene encoding the viral replicase and a capsid (VP) gene encoding the structural proteins of the virion [2]. The *Parvoviridae* family is divided into two subfamilies. All parvoviruses that infect vertebrates fall into one subfamily (*Parvovirinae*), which currently contains 41 viral species, classified into eight genera [1].

Parvoviruses cause disease in humans and domestic animals. For example, parvovirus B19—a species in the genus *Erythroparvovirus*—causes “erythema infectiosum” in children and polyarthropathy syndrome in adults [2], while canine parvovirus—a member of the genus *Protoparvovirus*—can cause haemorrhagic enteritis in dogs, with lethality in around 80% of cases [3].

In recent years, high throughput sequencing (HTS) approaches have been instrumental in the discovery of many novel parvovirus species [4,5,6,7]. Consequently, the known diversity of parvovirus species has expanded greatly and recent studies have suggested that the parvovirus host range may encompass the entire animal kingdom [8]. To understand the natural biology of vertebrate parvoviruses—that is, their dynamics in natural hosts, propensity to cause disease and zoonotic potential—it is important to document their distribution and diversity across a wide range of vertebrate species and populations. In this study, we used an HTS approach to investigate parvovirus infections among wild mammals and birds in Brazil.

## 2. Materials and Methods

### 2.1. Samples

**Figure 1:**
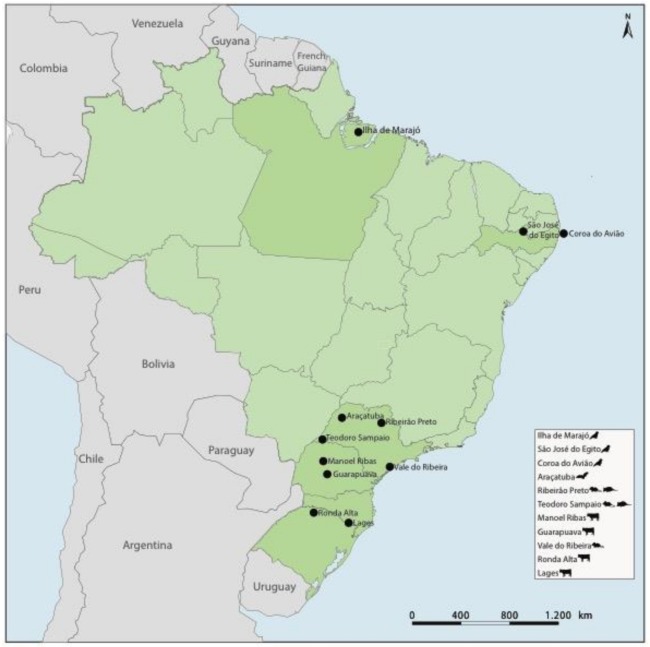
Geographic locations of collected samples in Brazil.

A total of 1073 specimens obtained from 21 different animal species were collected between 2007 and 2016 from rural areas of Pará, Pernambuco, São Paulo, Paraná, Santa Catarina and the Rio Grande do Sul states in Brazil. Individual specimens were distributed in 60 pools based on the species, sample type (i.e., tissue, blood, sera and cloacal swab), date and place of collection (Table S1). The species of wild animals were identified using morphological characteristics keys as previously described [9,10,11]. The geographical distribution of the pools is shown in Figure 1.

### 2.2. Preparation of Pools, Viral Genome Sequencing and Assembly

Tissue samples were individually homogenized with Hank’s balanced salt solution using the TissueLyser system (Qiagen, Germantown, MD, USA). Then, the homogenized tissue, sera and cloacal swabs were centrifuged for 5 min at 10,000 g and the pools were prepared as previously described [12]. The viral genomes were extracted with a QIAamp viral RNA mini kit (Qiagen, USA) and stored at −80 °C. Subsequently, the nucleic acid was quantified using a Qubit^®^ 2.0 Fluorometer (Invitrogen, Carlsbad, NM, USA) and the purity and integrity of nucleic acid of samples were measured using an Agilent 2100 Bioanalyzer (Agilent Technologies, Santa Clara, CA, USA). 

The ssDNAs were converted to dsDNA and sequenced in high-throughput sequencing using the RAPID module with the TruSeq RNA Universal kit (Illumina, San Diego, CA, USA) protocol and standard multiplex adaptors. A paired-end, 150-base-read protocol in the RAPID module was used for sequencing on an Illumina HiSeq 2500 instrument, as recommended by the manufacturer. Sequencing was performed at the Life Sciences Core Facility of the University of Campinas, Brazil. A total of 7,059,398 to 94,508,748 paired-end reads per pool were generated with 64.85% to 91.45% of bases ≥ Q30 with a base call accuracy of 99.9% (Table S1). The sequencing reads were assembled using the de novo approach in the metaViC pipeline (https://github.com/sejmodha/MetaViC) [12]. The parvovirus contigs longer than length of 200 nucleotides and supercontigs were merged and classified using DIAMOND against NCBI RefSeq protein database [13,14].

### 2.3. Genome Characterization

Genome size, coding potential and molecular protein weight were assessed with Geneious 9.1.2 (Biomatters, Auckland, New Zealand). The annotations of protein domains were performed using the Conserved Domain Database [15]. The nucleotide sequences determined in this study have been deposited in GenBank under the accession numbers listed in Table 1. 

### 2.4. Phylogenetic Analysis

Maximum likelihood (ML) phylogenetic trees were reconstructed using alignments of non-structural (NS) proteins and viral proteins (VPs), identified in the present study with representative members of the *Parvovirinae* subfamily [1]. Multiple sequence alignment (MSA) was carried out using RevTrans 2.0 [16] with manual adjustment. The alignments of the core of the NS and VP protein ML trees were inferred using IQ-TREE version 1.4.3 software based on an LG+F+G4 protein substitution model to the core of an NS protein with 145 amino acids and an LG+F+I+G4 protein substitution model to the core of a VP protein with 245 amino acids, both with 1000 replicates [17,18]. Statistical support for individual nodes was estimated via bootstrap replicates. Phylogenetic trees were visualized using Figtree 1.4.2 (http://tree.bio.ed.ac.uk/software/figtree/). Nucleotide divergence calculations were performed using the Sequence Demarcation Tool (SDT) version 1.2 in muscle mode [19].

## 3. Results

Using HTS, we identified 21 parvovirus sequences in samples derived from rodents, bats, opossums, birds and cattle in Pernambuco, São Paulo, Paraná and Rio Grande do Sul states in Brazil (Figure 1). These sequences comprised twelve nearly complete genomes and nine partial genomes (Table 1) and included the first examples of parvoviruses identified in opossums, New World bats and sigmondontine rodents. Parvovirus sequences recovered in our study were classified on the basis of (i) phylogeny and (ii) pairwise distance.

**Figure 2:**
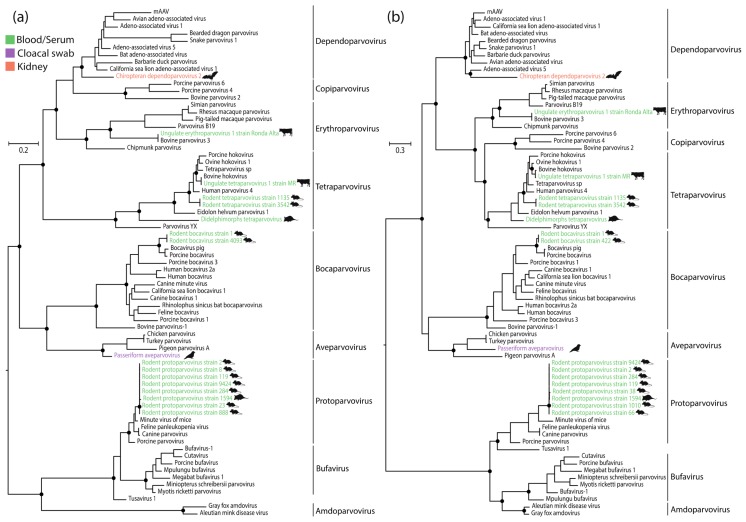
Maximum likelihood (ML) phylogenies showing the evolutionary relationships of newly identified parvoviruses. (**a**) Phylogenetic tree of non-structural (NS) proteins; (**b**) Phylogenetic tree of viral proteins (VPs). Phylogenies are midpoint rooted for clarity of presentation. The scale bar indicates evolutionary distance in substitutions per amino acid site. Black lines indicate genera within the *Parvovirinae* subfamily. Black circles indicate nodes with maximum likelihood bootstrap support levels >75%, based on 1000 bootstrap replicates. Taxa names of parvoviruses identified in our study are coloured according to sample type, as shown in the key. Silhouettes indicate host species groups.

To investigate the phylogenetic relationships between the novel parvoviruses and those described previously, we inferred ML phylogenetic trees from alignments of 71 NS proteins and 71 VP peptide sequences. Phylogenies revealed eight distinct clades corresponding to recognized genera, each having high bootstrap support (values > 75%). The sequences recovered in this study were grouped into six distinct genera (Figure 2). In most cases, the newly identified sequences grouped robustly within the established diversity of their respective genera. Only the *Dependoparvovirus*-like sequence identified in our study was grouped in a basal position with respect to previously characterized taxa in both NS and VP trees. 

According to the species demarcation criteria of the International Committee on Taxonomy of Viruses (ICTV), parvoviruses in the same species should share >85% amino acid sequence identity across the entire NS polypeptide sequence [1]. On this basis, the 21 genomes described in this study represent six novel species of parvoviruses and two that have been described previously—*Ungulate erythroparvovirus 1* and *ungulate tetraparvovirus 1* (Figures S1 and S2).

We identified a novel species of protoparvovirus in sigmondontine rodents. This virus, which was detected in samples from several distinct animals and species (Table 1), is quite similar to the minute virus of mice (MVM) but is sufficiently distinct based on ICTV criteria to be considered a distinct species. We also identified novel tetraparvoviruses in the opossum and hairy-tailed bolo mouse and a novel dependoparvovirus in tissue samples derived from common vampire bats (*Desmodus rotundus*). We identified a novel bocaparvovirus species—rodent bocaparvovirus—in two distinct sample pools obtained from hairy-tailed bolo mice and a novel aveparvovirus in the grey pileated finch in São José do Egito, Pernambuco State, Brazil. We also identified strains of two parvoviruses that were previously detected in cattle—*Ungulate erythroparvovirus 1* and *Ungulate tetraparvovirus 1*—identified in cattle serum of Ronda Alta in the Rio Grande do Sul State and Manoel Ribas in Paraná State, both located in South of Brazil.

**Figure 3:**
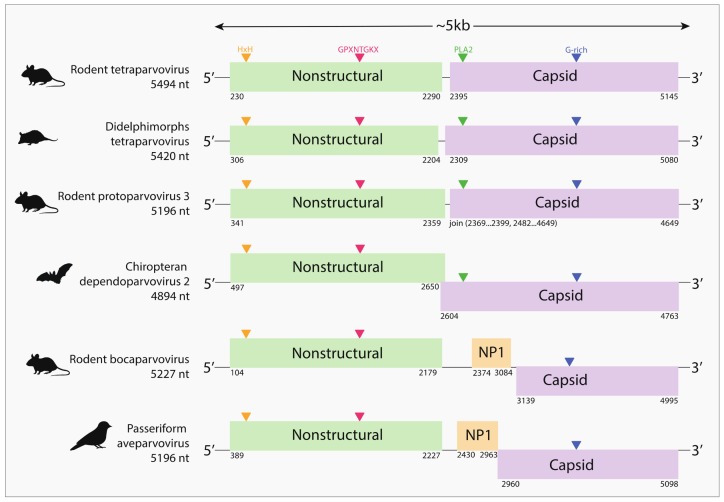
Genome structures of nearly complete coding sequences of newly identified parvoviruses. The length of the determined nucleotide sequences of the viral sequences are shown on the left. Boxes indicate the open reading frames (ORFs) and the number represents the respective position of their ORFs.

All the viruses identified in our study have typical parvovirus genome structures encoding NS and VP proteins. The deduced NS protein sequences from these viruses contain the “HxH” domain, which is similar to “HIH,” a metal binding domain previously described in the endonuclease domain [20,21]. This domain is a catalytic unit of the endonuclease, which was described to cleave one of the strands of dsDNA in viral cycle replication [2]. Also, we identified helicase motifs including Walker motifs [22], which are involved in viral DNA synthesis (Figure S3) [2,21]. Most of the capsid proteins also possess a glycine-rich (G-rich) region required for cellular entry [23] and the PLA_2_ motif involved in the viral release from the endosome and entry into the nucleus [24]. However, we did not identify a PLA_2_ motif in passeriform aveparvovirus or rodent bocaparvovirus. Interestingly, we observed that one species—chiropteran dependoparvovirus 2—encodes NS and VP as overlapping open reading frames (ORFs), with a shared region of 47 nucleotides (Figure 3). 

Notably, the rodent bocaparvoviruses and passeriform aveparvovirus contain a putative additional ORF (NP1). This gene is located in the middle of the viral genome and overlaps with the C-terminus region of the NS ORF but in a different reading frame (Figure 3). In the case of the rodent bocaparvoviruses, this ORF may correspond to the NP1 protein, which has been reported to play a role in efficient replication for human and canine bocaparvoviruses [25,26,27] and in immune evasion for porcine bocaparvoviruses [28]. 

## 4. Discussion

Brazil has a great diversity and abundance of wildlife and is considered a hotspot for the potential emergence of novel zoonotic viruses [29]. However, parvovirus studies in Brazil have focused predominantly on canine parvovirus and human parvovirus B19 [2,30]. In this study, we used an HTS approach to investigate parvovirus infections among wild mammals and birds from Brazil that were apparently without symptoms or disease. We identified 21 parvovirus sequences, representing six novel—and two previously described—parvovirus species. We report the first examples of parvoviruses in samples derived from *Sigmondontinae* rodents, opossums and New World bats. Interestingly, almost all the viruses detected here were sequenced from serum or blood samples suggesting that viremia may have been a factor in their identification.

We detected strains of *ungulate tetraparvovirus—*a virus in the genus *Tetraparvovirus*—in cattle from the South of Brazil. *Ungulate tetraparvovirus 2*—formerly known as porcine hokovirus—has previously been identified in swine in Brazil [31]. However, *ungulate tetraparvovirus 1*—formerly known as bovine hokovirus—has not previously been reported outside Asia. This virus, which was originally identified in bovine spleen samples obtained from food markets in Hong Kong, has also been identified in domestic yaks (*Bos grunniens*) in northwestern China [32,33]. The identification of this virus in an entirely distinct population (Brazilian cattle) not only establishes that it occurs outside Asia but also suggests it may be present in cattle populations throughout the world. In addition, we identified novel species of tetraparvovirus in samples obtained from rodents and from an opossum. Interestingly, the opossum sequence grouped basal relative to the largest *Tetraparvovirus* clade, which contains isolates from diverse eutherian mammals. Further sampling may reveal whether this basal position reflects the broad co-divergence of tetraparvoviruses and mammals dating back to the common ancestor of marsupials and eutherians. Such ancient origins of the *Tetraparvovirus* genus are consistent with evidence from endogenous viral element (EVE) sequences that parvoviruses have been infecting mammals for millions of years [34,35].

Recently, studies have reported numerous novel dependoparvoviruses in samples derived from Asian bats [36,37]. Here, we provide the first report of a dependoparvovirus in a New World bat—the vampire bat (*Desmodus rotundus*). In trees based on Rep, this virus groups basally within the *Dependoparvovirus* genus, consistent with these viruses potentially having an ancestral origin in bats, as has been proposed previously [36].

Currently, only one species is recognised in the genus *Aveparvovirus*. This virus (*Galliform aveparvovirus 1*) infects chickens and turkeys and is widespread in poultry farms in the United States and Europe [38,39]. We identified a novel *Aveparvovirus* species in samples derived from pileated finch (*Coryphospingus pileatus*), an indigenous (and non-migratory) South American bird, suggesting that viruses belonging to the *Aveparvovirus* genus may circulate widely among avian species, including wild as well as domestic birds.

We detected *Ungulate erythroparvovirus 1* (genus *Erythroparvovirus*) in Brazilian cattle. Since this virus—to the best of our knowledge—has only been described as a contaminant of commercial bovine serum [40], our study is the first to report detection of *Ungulate erythroparvovirus 1* in cattle populations.

We also identified a novel protoparvovirus species infecting sigmodontine rodents in Brazil. Sigmodontine rodent protoparvovirus was identified in several species of rodents (all belong to the subfamily) that we captured in the Ribeirão Preto region of São Paulo State. These viruses are closely related to the *Minute virus of mice* (MVM), a common pathogen of laboratory mice [41] but, following official taxonomic criteria, they are sufficiently divergent from MVM (>85% in NS and >73% aa in VP) to be considered a distinct species within the *Protoparvovirus* genus.

Bocaparvoviruses are associated with pathogenic conditions in human, bovine and canine hosts [2,42]. Rodent bocaparvoviruses have recently been reported [43] but relatively little is known about their broader distribution. We identified novel rodent bocaparvoviruses in sigmodontine rodents that are closely related to bocaparvoviruses recently reported in brown rats (*Rattus rattus*) in China [43] (data not shown). Together, these findings suggest a broad distribution for rodent bocaparvoviruses.

Parvoviruses that infect domestic and wild carnivores (including amdoviruses and protoparvoviruses) have been studied fairly extensively in the field. These studies have shown that groups of closely related parvoviruses circulate widely among species in the order Carnivora, with the barriers to transmission between species within the order apparently being relatively low [44,45,46]. The findings of our study suggest that this pattern might be reflected more broadly in parvovirus ecology, with many parvovirus genera containing sublineages that circulate within particular taxonomic groups of hosts (and are largely restricted to this host group). For example, the phylogenetic relationships shown in Figure 1 indicate that closely related protoparvoviruses circulate widely among rodents and that closely related tetraparvoviruses circulate widely in ungulates. With further sampling of parvovirus diversity, it should quickly become apparent whether these inferences are accurate. 

## 5. Conclusions

In this study, we used a sequencing-based approach to characterize parvovirus infections in wild and domestic animals in Brazil. Our findings extend the known biogeographic range of previously characterized parvovirus species and the known host range of three parvovirus genera (*Dependovirus*, *Aveparvovirus* and *Tetraparvovirus*). More broadly, our findings indicate that many parvovirus genera contain well-defined sub-lineages that circulate widely throughout the world within particular taxonomic groups of hosts. 

## Figures and Tables

**Table 1:** Sequences information, sources, sample, location, location, date and environment of viruses identified in wild animals from Brazil.

Genus	Viral Species	Strain	Genome	Size (nt)	Host Species	Sample	Samples Per Pool	Location	Date	GenBank
*Tetraparvovirus*	Rodent tetraparvovirus	1135	Nearly complete	5494	*Necromys lasiurus*	Blood	59	Ribeirão Preto, SP	2008	MG745669
*Tetraparvovirus*	Rodent tetraparvovirus	3542	Nearly complete	5494	*Necromys lasiurus*	Blood	52	Ribeirão Preto, SP	2009	MG745670
*Tetraparvovirus*	Didelphimorphs tetraparvovirus	4113	Nearly complete	5420	*Didelphis albiventris*	Serum	14	Teodoro Sampaio, SP	2009	MG745671
*Aveparvovirus*	Passeriform aveparvovirus	29	Nearly complete	5368	*Coryphospingus pileatus*	Cloacal Swab	4	São José do Egito, PE	2010	MG745672
*Bocaparvovirus*	Rodent bocaparvovirus	1	Nearly complete	5227	*Necromys lasiurus*	Blood	58	Ribeirão Preto, SP	2008	MG745673
*Protoparvovirus*	Rodent protoparvovirus	9424	Nearly complete	5219	*Necromys lasiurus*	Blood	58	Ribeirão Preto, SP	2008	MG745674
*Protoparvovirus*	Rodent protoparvovirus	284	Nearly complete	5196	*Akodon montensis*	Blood	41	Ribeirão Preto, SP	2009	MG745675
*Protoparvovirus*	Rodent protoparvovirus	119	Nearly complete	4998	*Calomys tener*	Blood	38	Ribeirão Preto, SP	2008	MG745676
*Dependoparvovirus*	Chiropteran dependoparvovirus 2	246	Nearly complete	4894	*Desmodus rotundus*	Kidney	8	Araçatuba, SP	2010	MG745677
*Protoparvovirus*	Rodent protoparvovirus	2	Nearly complete	4898	*Necromys lasiurus*	Blood	59	Ribeirão Preto, SP	2008	MG745678
*Tetraparvovirus*	Ungulate tetraparvovirus	MR	Nearly complete	5368	*Bos taurus*	Blood	15	Manoel Ribas, PR	2016	MG745679
*Erythroparvovirus*	Ungulate erythroparvovirus 1	Ronda Alta	Nearly complete	5220	*Bos taurus*	Blood	6	Ronda Alta, RS	2016	MG745680
*Protoparvovirus*	Rodent protoparvovirus	1594	Partial	2255	*Didelphis albiventris*	Blood	32	Ribeirão Preto, SP	2012–2013	MG745681
*Bocaparvovirus*	Rodent bocaparvovirus	4093	Partial	2844	*Necromys lasiurus*	Blood	52	Ribeirão Preto, SP	2009	MG745682
*Protoparvovirus*	Rodent protoparvovirus	8	Partial	1679	*Calomys tener*	Blood	34	Ribeirão Preto, SP	2009, 2012–2013	MG745683
*Protoparvovirus*	Rodent protoparvovirus	888	Partial	1606	*Oligoryzomys nigripes*	Blood	20	Ribeirão Preto, SP	2012–2013	MG745684
*Protoparvovirus*	Rodent protoparvovirus	23	Partial	1566	*Akodon montensis*	Blood	55	Ribeirão Preto, SP	2008	MG745685
*Bocaparvovirus*	Rodent bocaparvovirus	422	Partial	1362	*Necromys lasiurus*	Blood	52	Ribeirão Preto, SP	2009	MG745686
*Protoparvovirus*	Rodent protoparvovirus	1010	Partial	1283	*Oligoryzomys nigripes*	Blood	20	Ribeirão Preto, SP	2012–2013	MG745687
*Protoparvovirus*	Rodent protoparvovirus	66	Partial	1099	*Akodon montensis*	Blood	55	Ribeirão Preto, SP	2008	MG745688
*Protoparvovirus*	Rodent protoparvovirus	38	Partial	1067	*Calomys tener*	Blood	34	Ribeirão Preto, SP	2009,2012-2013	MG745689

Legend: SP (São Paulo State), PR (Paraná State), PE (Pernambuco State), RS (Rio Grande do Sul State).

## Supplementary Materials

The following are available online at http://www.mdpi.com/1999-4915/10/4/143/s1, Table S1: Samples information, host, sources, sample type, location, date, reads and %Bases ≥Q30. Figure S1: Heatmap of pairwise amino acid identities of the NS protein of parvoviruses identified in this study and representative members of the *Parvovirinae* subfamily based on ICTV criteria. The viruses described in this study are highlighted in bold. Figure S2: Heatmap of pairwise amino acid identities of the VP protein of parvoviruses identified in this study and representative members of the *Parvovirinae* subfamily based on ICTV criteria. The viruses described in this study are highlighted in bold. Figure S3: Alignment of helicase enzymatic motif showing walker’s motifs.
