# Supplementary material for: Novel Parvoviruses from Wild and Domestic Animals in Brazil Provide New Insights into Parvovirus Distribution and Diversity
Source: Viruses. 2018 Mar 22;10(4):143. doi: 10.3390/v10040143 (PMC5923437; doi:10.3390/v10040143)
Supplement: Supplementary file 1 [file viruses-10-00143-s001.zip › Supplementary Figure 1.pdf]

	Human parvovirus 4	Bovine hokovirus	**Ungulate tetraparvovirus**	Ovine hokovirus 1	Porcine hokovirus	Tetraparvovirus sp	**Rodent tetraparvovirus strain 1135**	**Rodent tetraparvovirus strain 3542**	Eidolon helvum parvovirus 1	Parvovirus YX	**Didelphimorphs tetraparvovirus**	Adeno-associated virus 1	Adeno-associated virus 5	California sea lion adeno-associated virus 1	Avian adeno-associated virus	Bat adeno-associated virus	Barbarie duck parvovirus	**Chiropteran dependoparvovirus 2**	Snake parvovirus 1	Bearded dragon parvovirus	Bovine parvovirus 2	Porcine parvovirus 4	Porcine parvovirus 6	Rhesus macaque parvovirus	Simian parvovirus	Pig-tailed macaque parvovirus	Human parvovirus B19	Bovine parvovirus 3	**Ungulate erythroparvovirus 1**	Chipmunk parvovirus	Bovine parvovirus 1	Canine minute virus	California sea lion bocavirus 1	Canine bocavirus 1	Feline bocavirus	Bocavirus pig	Porcine bocavirus	**Rodent bocaparvovirus strain 1**	**Rodent bocaparvovirus strain 4093**	Porcine bocavirus 1	Rhinolophus sinicus bat bocaparvovirus	Human bocavirus 2a	Human bocavirus	Porcine bocavirus 3	Chicken parvovirus	Turkey parvovirus	**Passeriform aveparvovirus**	Pigeon parvovirus A	Feline panleukopenia virus	Canine parvovirus	Minute virus of mice	**Rodent protoparvovirus strain 2**	**Rodent protoparvovirus strain 23**	**Rodent protoparvovirus strain 119**	**Rodent protoparvovirus strain 9424**	**Rodent protoparvovirus strain 284**	**Rodent protoparvovirus strain 8**	**Rodent protoparvovirus strain 1594**	Porcine parvovirus	Tusavirus 1	Bufavirus 1	Cutavirus	Myotis ricketti parvovirus	Miniopterus schreibersii parvovirus	Porcine bufavirus	Mpulungu bufavirus	Megabat bufavirus 1	Aleutian mink disease virus	Gray fox amdovirus
Human parvovirus 4	1.00
Bovine hokovirus	0.60	1.00
**Ungulate tetraparvovirus**	0.60	0.97	1.00
Ovine hokovirus 1	0.61	0.76	0.78	1.00
Porcine hokovirus	0.60	0.70	0.71	0.71	1.00
Tetraparvovirus sp	0.59	0.70	0.70	0.70	0.66	1.00
**Rodent tetraparvovirus strain 1135**	0.57	0.55	0.55	0.57	0.54	0.57	1.00
**Rodent tetraparvovirus strain 3542**	0.56	0.56	0.54	0.57	0.54	0.57	0.98	1.00
Eidolon helvum parvovirus 1	0.50	0.50	0.51	0.49	0.52	0.50	0.48	0.48	1.00
Parvovirus YX	0.39	0.39	0.40	0.41	0.41	0.39	0.39	0.40	0.38	1.00
**Didelphimorphs tetraparvovirus**	0.42	0.43	0.43	0.40	0.42	0.41	0.42	0.42	0.40	0.42	1.00
Adeno-associated virus 1	0.31	0.31	0.31	0.30	0.32	0.31	0.32	0.32	0.33	0.30	0.29	1.00
Adeno-associated virus 5	0.29	0.33	0.33	0.31	0.31	0.31	0.32	0.31	0.32	0.30	0.29	0.64	1.00
California sea lion adeno-associated virus 1	0.33	0.31	0.31	0.32	0.33	0.34	0.32	0.31	0.34	0.31	0.30	0.59	0.65	1.00
Avian adeno-associated virus	0.30	0.31	0.30	0.31	0.32	0.30	0.30	0.30	0.30	0.29	0.31	0.58	0.54	0.53	1.00
Bat adeno-associated virus	0.30	0.32	0.33	0.31	0.31	0.32	0.33	0.33	0.34	0.31	0.27	0.55	0.54	0.54	0.50	1.00
Barbarie duck parvovirus	0.31	0.31	0.31	0.32	0.32	0.30	0.33	0.32	0.32	0.29	0.31	0.50	0.51	0.49	0.51	0.46	1.00
**Chiropteran dependoparvovirus 2**	0.36	0.36	0.36	0.36	0.36	0.37	0.33	0.32	0.31	0.30	0.33	0.44	0.44	0.46	0.42	0.44	0.47	1.00
Snake parvovirus 1	0.32	0.32	0.30	0.31	0.31	0.31	0.32	0.33	0.32	0.31	0.28	0.42	0.41	0.39	0.42	0.42	0.40	0.37	1.00
Bearded dragon parvovirus	0.33	0.30	0.31	0.32	0.31	0.30	0.32	0.31	0.33	0.30	0.29	0.41	0.41	0.39	0.39	0.38	0.37	0.37	0.59	1.00
Bovine parvovirus 2	0.32	0.31	0.30	0.32	0.30	0.30	0.31	0.31	0.30	0.29	0.27	0.32	0.34	0.33	0.33	0.33	0.32	0.33	0.29	0.31	1.00
Porcine parvovirus 4	0.32	0.30	0.29	0.32	0.29	0.30	0.31	0.31	0.28	0.31	0.28	0.33	0.35	0.32	0.31	0.34	0.31	0.34	0.31	0.32	0.38	1.00
Porcine parvovirus 6	0.32	0.34	0.31	0.31	0.30	0.32	0.30	0.30	0.30	0.31	0.31	0.36	0.36	0.35	0.33	0.35	0.37	0.34	0.32	0.31	0.37	0.59	1.00
Rhesus macaque parvovirus	0.28	0.30	0.29	0.30	0.28	0.27	0.28	0.28	0.28	0.26	0.28	0.33	0.34	0.35	0.30	0.34	0.32	0.31	0.32	0.29	0.30	0.29	0.28	1.00
Simian parvovirus	0.31	0.31	0.30	0.29	0.30	0.29	0.29	0.29	0.27	0.27	0.29	0.34	0.31	0.34	0.31	0.33	0.32	0.32	0.30	0.30	0.30	0.31	0.29	0.79	1.00
Pig-tailed macaque parvovirus	0.28	0.27	0.27	0.29	0.31	0.30	0.29	0.29	0.28	0.28	0.29	0.34	0.32	0.34	0.31	0.32	0.30	0.31	0.32	0.33	0.30	0.31	0.29	0.66	0.69	1.00
Human parvovirus B19	0.29	0.28	0.28	0.28	0.30	0.30	0.29	0.28	0.28	0.30	0.28	0.31	0.29	0.33	0.29	0.33	0.29	0.31	0.29	0.30	0.33	0.28	0.25	0.44	0.44	0.45	1.00
Bovine parvovirus 3	0.30	0.30	0.31	0.30	0.33	0.31	0.32	0.33	0.31	0.32	0.31	0.31	0.31	0.31	0.29	0.31	0.30	0.31	0.31	0.31	0.29	0.30	0.29	0.34	0.37	0.33	0.35	1.00
**Ungulate erythroparvovirus 1**	0.32	0.31	0.32	0.32	0.31	0.31	0.32	0.32	0.31	0.32	0.30	0.30	0.31	0.29	0.29	0.31	0.30	0.31	0.31	0.30	0.29	0.31	0.30	0.33	0.35	0.32	0.34	0.99	1.00
Chipmunk parvovirus	0.31	0.30	0.31	0.29	0.32	0.30	0.30	0.31	0.30	0.29	0.29	0.32	0.33	0.37	0.31	0.36	0.32	0.34	0.30	0.29	0.31	0.29	0.28	0.29	0.29	0.32	0.35	0.38	0.40	1.00
Bovine parvovirus 1	0.27	0.25	0.25	0.24	0.27	0.25	0.29	0.29	0.26	0.25	0.26	0.29	0.28	0.30	0.26	0.30	0.27	0.28	0.27	0.28	0.27	0.27	0.28	0.27	0.27	0.27	0.27	0.29	0.30	0.29	1.00
Canine minute virus	0.27	0.28	0.26	0.25	0.26	0.26	0.28	0.27	0.27	0.24	0.25	0.28	0.29	0.28	0.27	0.28	0.27	0.28	0.29	0.28	0.26	0.29	0.27	0.26	0.25	0.27	0.28	0.27	0.27	0.27	0.37	1.00
California sea lion bocavirus 1	0.27	0.27	0.27	0.26	0.29	0.27	0.27	0.27	0.24	0.26	0.27	0.26	0.27	0.27	0.27	0.30	0.26	0.29	0.31	0.29	0.29	0.28	0.28	0.27	0.26	0.26	0.26	0.28	0.28	0.27	0.37	0.55	1.00
Canine bocavirus 1	0.30	0.27	0.26	0.25	0.27	0.26	0.29	0.29	0.27	0.26	0.26	0.29	0.29	0.28	0.27	0.28	0.28	0.30	0.28	0.27	0.29	0.27	0.26	0.29	0.30	0.30	0.29	0.28	0.29	0.28	0.41	0.60	0.66	1.00
Feline bocavirus	0.26	0.28	0.27	0.25	0.26	0.26	0.28	0.24	0.27	0.24	0.27	0.29	0.28	0.29	0.27	0.29	0.27	0.28	0.30	0.29	0.30	0.28	0.26	0.26	0.27	0.27	0.28	0.26	0.27	0.26	0.35	0.52	0.52	0.56	1.00
Bocavirus pig	0.29	0.25	0.25	0.26	0.26	0.26	0.27	0.26	0.27	0.25	0.25	0.28	0.29	0.31	0.30	0.28	0.29	0.30	0.28	0.29	0.29	0.29	0.28	0.27	0.29	0.31	0.32	0.30	0.28	0.29	0.38	0.48	0.49	0.47	0.48	1.00
Porcine bocavirus	0.29	0.26	0.26	0.26	0.26	0.26	0.26	0.27	0.28	0.25	0.28	0.29	0.27	0.29	0.28	0.28	0.26	0.29	0.28	0.28	0.30	0.26	0.28	0.28	0.28	0.29	0.32	0.28	0.28	0.27	0.38	0.48	0.47	0.45	0.48	0.81	1.00
**Rodent bocaparvovirus strain 1**	0.29	0.28	0.27	0.28	0.28	0.28	0.29	0.29	0.28	0.27	0.25	0.29	0.30	0.29	0.30	0.30	0.29	0.29	0.31	0.29	0.29	0.28	0.28	0.29	0.28	0.28	0.29	0.27	0.28	0.29	0.38	0.46	0.47	0.46	0.47	0.76	0.77	1.00
**Rodent bocaparvovirus strain 4093**	0.29	0.28	0.27	0.28	0.28	0.28	0.29	0.29	0.28	0.27	0.25	0.29	0.30	0.29	0.30	0.30	0.29	0.29	0.31	0.29	0.29	0.28	0.28	0.29	0.28	0.28	0.29	0.27	0.28	0.29	0.38	0.46	0.47	0.46	0.47	0.76	0.77	1.00	1.00
Porcine bocavirus 1	0.30	0.27	0.27	0.28	0.28	0.30	0.28	0.27	0.27	0.25	0.26	0.28	0.27	0.28	0.26	0.29	0.27	0.30	0.29	0.29	0.30	0.27	0.29	0.28	0.31	0.30	0.33	0.28	0.27	0.31	0.37	0.45	0.47	0.51	0.48	0.47	0.48	0.47	0.47	1.00
Rhinolophus sinicus bat bocaparvovirus	0.28	0.30	0.31	0.27	0.28	0.29	0.25	0.26	0.28	0.26	0.24	0.32	0.27	0.29	0.27	0.30	0.25	0.28	0.27	0.27	0.30	0.25	0.27	0.29	0.28	0.28	0.31	0.30	0.30	0.32	0.40	0.46	0.46	0.48	0.46	0.43	0.41	0.43	0.43	0.46	1.00
Human bocavirus 2a	0.28	0.25	0.25	0.26	0.26	0.27	0.27	0.27	0.28	0.26	0.27	0.29	0.29	0.30	0.27	0.31	0.29	0.29	0.28	0.28	0.25	0.26	0.29	0.30	0.30	0.30	0.30	0.29	0.27	0.32	0.40	0.44	0.46	0.46	0.44	0.42	0.42	0.41	0.41	0.43	0.42	1.00
Human bocavirus	0.27	0.24	0.25	0.25	0.27	0.26	0.27	0.27	0.28	0.25	0.24	0.28	0.29	0.28	0.27	0.27	0.27	0.28	0.29	0.26	0.26	0.27	0.29	0.31	0.31	0.29	0.31	0.29	0.27	0.28	0.40	0.45	0.46	0.45	0.46	0.43	0.42	0.42	0.42	0.44	0.43	0.77	1.00
Porcine bocavirus 3	0.26	0.27	0.26	0.25	0.27	0.24	0.30	0.30	0.27	0.27	0.26	0.29	0.27	0.27	0.28	0.29	0.29	0.29	0.25	0.24	0.31	0.27	0.30	0.29	0.31	0.27	0.30	0.31	0.31	0.29	0.41	0.41	0.43	0.41	0.41	0.43	0.44	0.43	0.43	0.43	0.40	0.44	0.41	1.00
Chicken parvovirus	0.25	0.27	0.29	0.26	0.25	0.26	0.26	0.26	0.25	0.27	0.25	0.25	0.25	0.28	0.26	0.29	0.25	0.28	0.26	0.26	0.29	0.27	0.26	0.24	0.24	0.26	0.25	0.26	0.27	0.29	0.27	0.25	0.26	0.28	0.27	0.28	0.27	0.27	0.27	0.28	0.27	0.29	0.30	0.28	1.00
Turkey parvovirus	0.25	0.29	0.29	0.26	0.26	0.27	0.27	0.26	0.25	0.27	0.25	0.25	0.25	0.28	0.26	0.29	0.26	0.29	0.26	0.26	0.28	0.28	0.26	0.25	0.25	0.26	0.26	0.27	0.27	0.24	0.28	0.25	0.27	0.29	0.27	0.28	0.27	0.27	0.27	0.28	0.27	0.28	0.30	0.29	0.99	1.00
**Passeriform aveparvovirus**	0.26	0.28	0.29	0.28	0.26	0.29	0.28	0.28	0.26	0.29	0.26	0.30	0.29	0.28	0.28	0.31	0.28	0.31	0.26	0.30	0.28	0.28	0.30	0.28	0.28	0.30	0.30	0.28	0.27	0.30	0.29	0.28	0.31	0.30	0.30	0.31	0.30	0.30	0.30	0.30	0.27	0.29	0.31	0.30	0.54	0.54	1.00
Pigeon parvovirus A	0.26	0.27	0.28	0.26	0.27	0.27	0.28	0.28	0.28	0.27	0.24	0.26	0.28	0.28	0.27	0.28	0.29	0.31	0.29	0.28	0.28	0.25	0.27	0.26	0.26	0.29	0.29	0.29	0.27	0.29	0.28	0.28	0.28	0.25	0.28	0.28	0.29	0.29	0.29	0.29	0.24	0.28	0.27	0.29	0.46	0.47	0.47	1.00
Feline panleukopenia virus	0.23	0.23	0.23	0.23	0.24	0.23	0.24	0.24	0.22	0.25	0.25	0.26	0.27	0.27	0.28	0.29	0.26	0.27	0.28	0.27	0.26	0.25	0.23	0.26	0.25	0.26	0.25	0.27	0.26	0.25	0.25	0.27	0.27	0.25	0.24	0.28	0.28	0.28	0.28	0.25	0.21	0.29	0.26	0.27	0.22	0.22	0.25	0.24	1.00
Canine parvovirus	0.23	0.23	0.23	0.23	0.24	0.23	0.24	0.24	0.22	0.25	0.25	0.27	0.27	0.27	0.28	0.29	0.26	0.27	0.28	0.27	0.26	0.25	0.23	0.26	0.25	0.26	0.25	0.27	0.26	0.25	0.25	0.27	0.27	0.26	0.24	0.27	0.27	0.27	0.27	0.24	0.21	0.29	0.26	0.28	0.21	0.22	0.26	0.25	0.99	1.00
Minute virus of mice	0.25	0.24	0.23	0.22	0.24	0.24	0.23	0.24	0.24	0.24	0.23	0.27	0.27	0.27	0.26	0.29	0.27	0.27	0.27	0.27	0.29	0.26	0.27	0.27	0.26	0.25	0.25	0.28	0.27	0.26	0.25	0.26	0.27	0.27	0.25	0.28	0.29	0.28	0.28	0.26	0.23	0.28	0.27	0.29	0.23	0.23	0.25	0.26	0.74	0.74	1.00
**Rodent protoparvovirus strain 2**	0.23	0.23	0.23	0.24	0.21	0.23	0.23	0.23	0.23	0.24	0.23	0.27	0.29	0.26	0.27	0.28	0.27	0.28	0.28	0.26	0.26	0.25	0.26	0.27	0.27	0.26	0.24	0.27	0.26	0.28	0.27	0.26	0.26	0.26	0.25	0.27	0.29	0.27	0.27	0.24	0.24	0.29	0.28	0.28	0.25	0.24	0.26	0.26	0.73	0.72	0.84	1.00
**Rodent protoparvovirus strain 23**	0.17	0.20	0.16	0.22	0.22	0.20	0.19	0.18	0.22	0.22	0.20	0.24	0.22	0.22	0.19	0.22	0.26	0.22	0.28	0.26	0.26	0.23	0.20	0.20	0.23	0.21	0.21	0.18	0.24	0.18	0.23	0.25	0.24	0.25	0.23	0.25	0.27	0.24	0.24	0.23	0.23	0.26	0.28	0.26	0.26	0.26	0.24	0.23	0.73	0.73	0.84	1.00	1.00
**Rodent protoparvovirus strain 119**	0.23	0.23	0.23	0.24	0.21	0.23	0.23	0.23	0.23	0.24	0.23	0.27	0.29	0.26	0.27	0.28	0.27	0.28	0.28	0.26	0.26	0.25	0.26	0.27	0.27	0.26	0.24	0.27	0.26	0.28	0.27	0.26	0.26	0.26	0.25	0.27	0.29	0.27	0.27	0.24	0.24	0.29	0.28	0.28	0.25	0.24	0.26	0.26	0.73	0.72	0.84	1.00	1.00	1.00
**Rodent protoparvovirus strain 9424**	0.23	0.23	0.23	0.24	0.21	0.23	0.23	0.23	0.23	0.24	0.23	0.27	0.29	0.26	0.27	0.28	0.27	0.28	0.28	0.26	0.26	0.25	0.26	0.27	0.27	0.26	0.24	0.27	0.26	0.28	0.27	0.26	0.26	0.26	0.24	0.27	0.29	0.27	0.27	0.24	0.24	0.29	0.28	0.28	0.25	0.24	0.26	0.26	0.73	0.72	0.84	1.00	1.00	1.00	1.00
**Rodent protoparvovirus strain 284**	0.23	0.24	0.24	0.24	0.21	0.23	0.24	0.24	0.22	0.24	0.24	0.27	0.27	0.26	0.27	0.28	0.28	0.29	0.28	0.26	0.26	0.24	0.25	0.27	0.27	0.27	0.26	0.27	0.25	0.28	0.27	0.26	0.26	0.26	0.26	0.27	0.29	0.27	0.27	0.24	0.24	0.29	0.28	0.29	0.25	0.25	0.26	0.26	0.73	0.73	0.84	0.99	0.99	0.99	0.99	1.00
**Rodent protoparvovirusstrain 8**	0.24	0.26	0.26	0.25	0.24	0.25	0.24	0.24	0.26	0.25	0.27	0.28	0.27	0.27	0.26	0.28	0.26	0.29	0.26	0.24	0.25	0.23	0.25	0.26	0.27	0.27	0.26	0.28	0.29	0.25	0.28	0.28	0.26	0.25	0.27	0.28	0.31	0.30	0.30	0.27	0.26	0.31	0.29	0.29	0.25	0.24	0.25	0.27	0.73	0.73	0.82	0.95	1.00	0.95	0.95	0.95	1.00
**Rodent protoparvovirus strain 1594**	0.29	0.31	0.32	0.32	0.28	0.30	0.28	0.28	0.28	0.30	0.26	0.35	0.40	0.33	0.35	0.33	0.32	0.34	0.34	0.32	0.32	0.33	0.33	0.33	0.34	0.31	0.31	0.35	0.34	0.35	0.35	0.33	0.32	0.34	0.33	0.31	0.36	0.37	0.37	0.29	0.31	0.39	0.35	0.33	0.28	0.28	0.32	0.35	0.75	0.75	0.85	1.00	0.23	1.00	1.00	0.98	0.89	1.00
Porcine parvovirus	0.26	0.25	0.24	0.25	0.26	0.27	0.25	0.26	0.23	0.27	0.21	0.28	0.26	0.26	0.28	0.29	0.27	0.27	0.25	0.27	0.26	0.26	0.26	0.27	0.27	0.28	0.26	0.27	0.26	0.28	0.25	0.28	0.26	0.25	0.26	0.28	0.28	0.28	0.28	0.24	0.26	0.29	0.28	0.27	0.23	0.23	0.23	0.25	0.67	0.67	0.70	0.68	0.70	0.68	0.68	0.68	0.69	0.70	1.00
Tusavirus 1	0.28	0.27	0.26	0.27	0.25	0.26	0.27	0.27	0.24	0.25	0.24	0.27	0.29	0.27	0.28	0.30	0.29	0.28	0.28	0.26	0.26	0.26	0.26	0.28	0.26	0.27	0.26	0.29	0.25	0.28	0.25	0.27	0.28	0.25	0.28	0.24	0.27	0.27	0.27	0.25	0.27	0.29	0.29	0.29	0.25	0.25	0.23	0.26	0.47	0.47	0.48	0.48	0.41	0.48	0.48	0.48	0.49	0.60	0.47	1.00
Bufavirus 1	0.25	0.24	0.25	0.25	0.24	0.25	0.27	0.26	0.24	0.26	0.22	0.29	0.27	0.28	0.27	0.29	0.28	0.27	0.27	0.25	0.25	0.23	0.28	0.24	0.26	0.26	0.26	0.27	0.26	0.26	0.25	0.23	0.24	0.24	0.24	0.21	0.24	0.23	0.23	0.23	0.22	0.26	0.25	0.26	0.24	0.24	0.25	0.26	0.41	0.41	0.43	0.42	0.40	0.42	0.42	0.42	0.45	0.49	0.44	0.46	1.00
Cutavirus	0.24	0.22	0.23	0.25	0.23	0.24	0.27	0.27	0.23	0.24	0.20	0.26	0.26	0.28	0.27	0.28	0.26	0.26	0.26	0.26	0.23	0.24	0.25	0.25	0.24	0.26	0.26	0.25	0.23	0.24	0.22	0.26	0.26	0.24	0.26	0.26	0.25	0.24	0.24	0.24	0.23	0.26	0.25	0.25	0.25	0.25	0.24	0.26	0.43	0.43	0.43	0.43	0.40	0.43	0.43	0.43	0.43	0.51	0.43	0.46	0.77	1.00
Myotis ricketti parvovirus	0.28	0.28	0.28	0.28	0.29	0.27	0.28	0.28	0.27	0.25	0.27	0.27	0.27	0.28	0.27	0.28	0.27	0.28	0.25	0.26	0.24	0.23	0.27	0.25	0.25	0.26	0.28	0.26	0.25	0.26	0.28	0.31	0.28	0.28	0.29	0.27	0.27	0.29	0.29	0.27	0.28	0.29	0.26	0.28	0.26	0.26	0.25	0.24	0.49	0.49	0.50	0.48	0.48	0.48	0.48	0.49	0.48	0.55	0.50	0.48	0.60	0.58	1.00
Miniopterus schreibersii parvovirus	0.29	0.29	0.29	0.31	0.29	0.28	0.29	0.30	0.28	0.28	0.25	0.30	0.30	0.30	0.30	0.31	0.30	0.32	0.27	0.28	0.27	0.26	0.29	0.25	0.28	0.29	0.27	0.31	0.30	0.29	0.27	0.35	0.29	0.30	0.28	0.28	0.30	0.28	0.28	0.28	0.28	0.27	0.29	0.29	0.28	0.28	0.27	0.27	0.50	0.50	0.54	0.50	0.47	0.50	0.50	0.49	0.50	0.54	0.53	0.49	0.63	0.63	0.81	1.00
Porcine bufavirus	0.25	0.24	0.25	0.27	0.26	0.25	0.24	0.28	0.24	0.27	0.24	0.29	0.27	0.27	0.26	0.29	0.26	0.28	0.26	0.25	0.23	0.27	0.26	0.24	0.28	0.27	0.29	0.26	0.24	0.26	0.30	0.28	0.26	0.29	0.27	0.28	0.29	0.28	0.28	0.26	0.24	0.31	0.29	0.29	0.29	0.28	0.26	0.25	0.47	0.48	0.50	0.45	0.44	0.45	0.45	0.47	0.46	0.50	0.47	0.46	0.61	0.61	0.63	0.64	1.00
Mpulungu bufavirus	0.27	0.26	0.26	0.27	0.23	0.26	0.30	0.27	0.26	0.25	0.26	0.23	0.29	0.26	0.26	0.29	0.26	0.27	0.27	0.26	0.24	0.26	0.26	0.25	0.26	0.25	0.29	0.27	0.25	0.25	0.25	0.27	0.27	0.25	0.25	0.24	0.25	0.26	0.26	0.26	0.26	0.28	0.27	0.26	0.24	0.24	0.23	0.25	0.43	0.43	0.45	0.41	0.36	0.41	0.41	0.41	0.44	0.55	0.44	0.44	0.56	0.57	0.62	0.63	0.66	1.00
Megabat bufavirus 1	0.26	0.23	0.25	0.25	0.24	0.24	0.26	0.26	0.26	0.25	0.24	0.28	0.27	0.28	0.28	0.30	0.28	0.26	0.27	0.24	0.25	0.25	0.29	0.26	0.27	0.27	0.26	0.24	0.24	0.26	0.26	0.28	0.28	0.24	0.26	0.24	0.25	0.26	0.26	0.23	0.25	0.27	0.26	0.30	0.26	0.25	0.25	0.23	0.45	0.44	0.44	0.43	0.39	0.43	0.43	0.43	0.45	0.55	0.44	0.45	0.56	0.56	0.57	0.60	0.61	0.55	1.00
Aleutian mink disease virus	0.24	0.24	0.24	0.23	0.22	0.25	0.24	0.24	0.22	0.27	0.25	0.27	0.28	0.28	0.28	0.28	0.27	0.28	0.25	0.27	0.25	0.24	0.26	0.27	0.26	0.27	0.26	0.29	0.26	0.24	0.26	0.25	0.26	0.26	0.23	0.24	0.26	0.26	0.26	0.24	0.23	0.25	0.24	0.26	0.25	0.24	0.27	0.24	0.30	0.30	0.31	0.30	0.26	0.30	0.30	0.30	0.30	0.39	0.30	0.29	0.27	0.27	0.30	0.31	0.30	0.29	0.29	1.00
Gray fox amdovirus	0.27	0.23	0.25	0.23	0.22	0.26	0.25	0.25	0.24	0.27	0.23	0.30	0.27	0.28	0.24	0.25	0.26	0.28	0.25	0.24	0.26	0.24	0.25	0.26	0.27	0.27	0.27	0.25	0.25	0.24	0.28	0.23	0.26	0.24	0.23	0.24	0.25	0.23	0.23	0.25	0.23	0.24	0.25	0.25	0.26	0.26	0.27	0.26	0.28	0.28	0.28	0.28	0.24	0.28	0.28	0.27	0.28	0.40	0.28	0.28	0.27	0.28	0.32	0.34	0.33	0.30	0.30	0.69	1.00
