# Supplementary figures and images for: Novel Parvoviruses from Wild and Domestic Animals in Brazil Provide New Insights into Parvovirus Distribution and Diversity
Source: Viruses. 2018 Mar 22;10(4):143. doi: 10.3390/v10040143 (PMC5923437; doi:10.3390/v10040143)

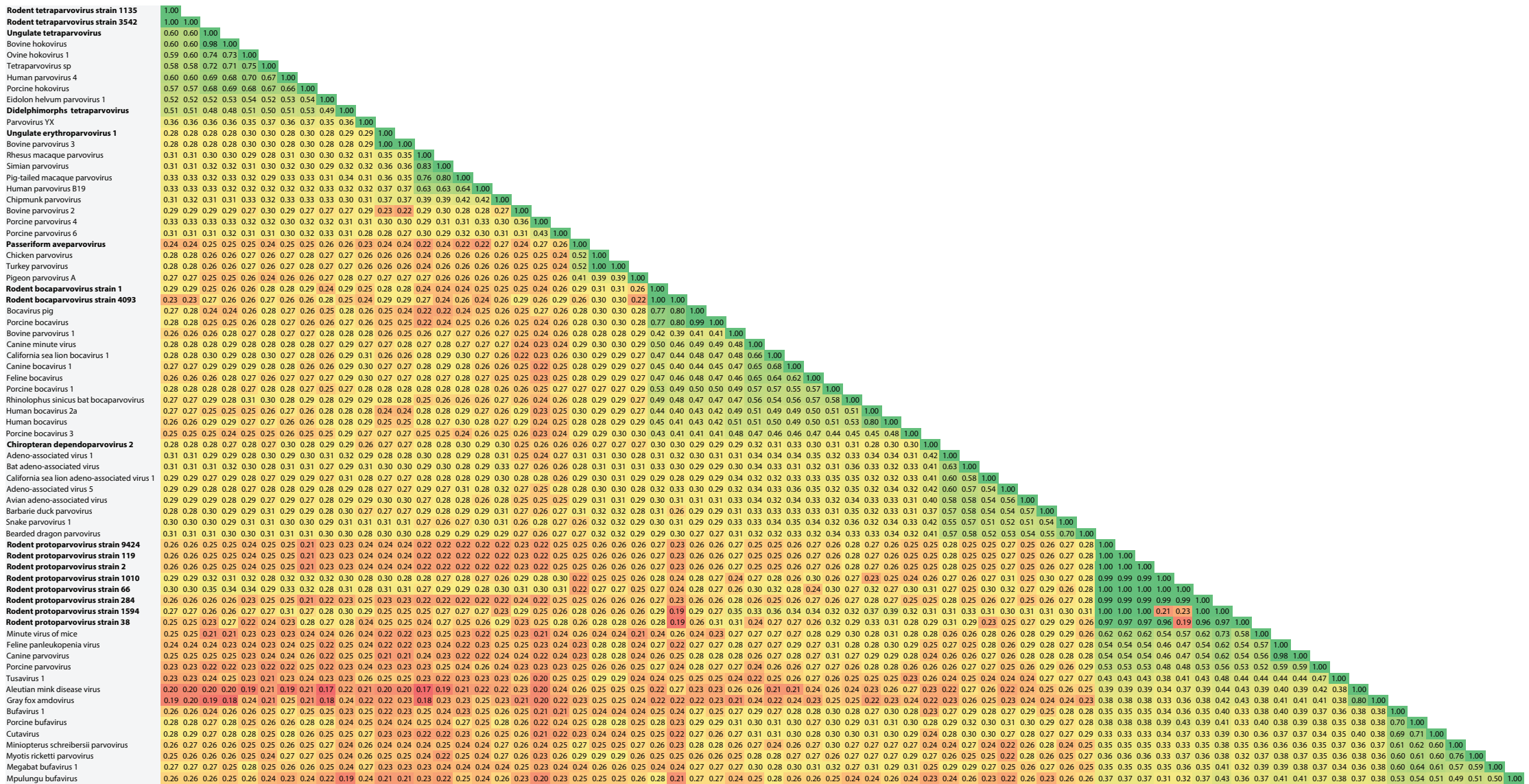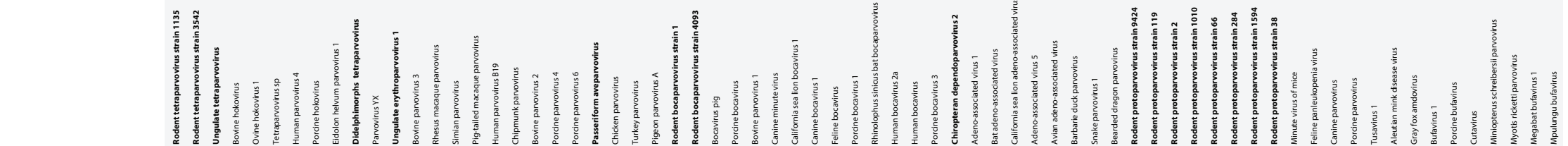

Supplement: Supplementary file 1 [file viruses-10-00143-s001.zip › Supplementary Figure 2.pdf]

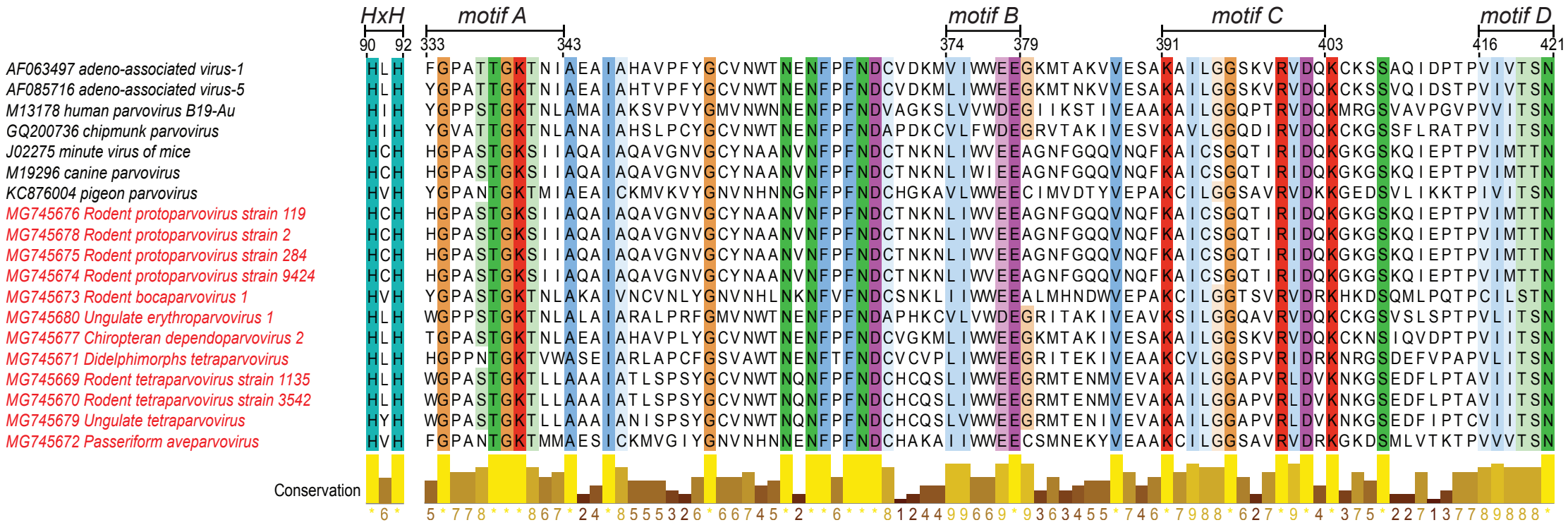

Supplement: Supplementary file 1 [file viruses-10-00143-s001.zip › Supplementary Figure 3.pdf]
